# Selenium Nanoparticles in Protecting the Brain from Stroke: Possible Signaling and Metabolic Mechanisms

**DOI:** 10.3390/nano14020160

**Published:** 2024-01-11

**Authors:** Egor A. Turovsky, Alexey S. Baryshev, Egor Y. Plotnikov

**Affiliations:** 1 Institute of Cell Biophysics of the Russian Academy of Sciences, Federal Research Center “Pushchino Scientific Center for Biological Research of the Russian Academy of Sciences”, 142290 Pushchino, Russia; 2 Prokhorov General Physics Institute of the Russian Academy of Sciences, 38 Vavilove st., 119991 Moscow, Russia; aleksej.baryshev@gmail.com; 3 A.N. Belozersky Institute of Physico-Chemical Biology, Lomonosov Moscow State University, 119992 Moscow, Russia

**Keywords:** nanomaterials, selenium, selenium nanoparticles, stroke, neuroprotection, signaling

## Abstract

Strokes rank as the second most common cause of mortality and disability in the human population across the world. Currently, available methods of treating or preventing strokes have significant limitations, primarily the need to use high doses of drugs due to the presence of the blood–brain barrier. In the last decade, increasing attention has been paid to the capabilities of nanotechnology. However, the vast majority of research in this area is focused on the mechanisms of anticancer and antiviral effects of nanoparticles. In our opinion, not enough attention is paid to the neuroprotective mechanisms of nanomaterials. In this review, we attempted to summarize the key molecular mechanisms of brain cell damage during ischemia. We discussed the current literature regarding the use of various nanomaterials for the treatment of strokes. In this review, we examined the features of all known nanomaterials, the possibility of which are currently being studied for the treatment of strokes. In this regard, the positive and negative properties of nanomaterials for the treatment of strokes have been identified. Particular attention in the review was paid to nanoselenium since selenium is a vital microelement and is part of very important and little-studied proteins, e.g., selenoproteins and selenium-containing proteins. An analysis of modern studies of the cytoprotective effects of nanoselenium made it possible to establish the mechanisms of acute and chronic protective effects of selenium nanoparticles. In this review, we aimed to combine all the available information regarding the neuroprotective properties and mechanisms of action of nanoparticles in neurodegenerative processes, especially in cerebral ischemia.

## 1. Introduction

A large number of people in all countries of the world die or become disabled every year from ischemic stroke. Strokes are responsible for 24.9% of lifetime mortality (18.3% for ischemic stroke and 8.2% for hemorrhagic stroke) in people over 25 years of age [1,2]. The causes of strokes include thrombosis or embolism, as well as atherosclerosis and aneurysms in the case of hemorrhagic stroke [3,4]. For the treatment of strokes, three approaches are mainly used in the clinic: the application of intravenous thrombolytics [5], primarily alteplase (recombinant tissue plasminogen activator, tPA), and endovascular thrombectomy [6]. Moreover, in the subset of strokes with emergent large vessel occlusion, tPA is less effective due to its limited contact and penetration within a large clot to cause thrombolysis and recanalization due to its short half-life (~5 min) [7]. Rapid restoration of blood flow (reperfusion) remains the preferred treatment for reducing the area of brain damage after a stroke. However, 2/3 of people who have had a stroke remain significantly disabled. At the same time, reperfusion of the ischemic area also has devastating consequences, contributing to increased tissue damage with further worsening of the disease outcome due to the induction of oxidative stress [1]. Current options for treating and preventing strokes remain limited. The blood–brain barrier (BBB) is a key issue for stroke treatment. The blood–brain barrier is formed by vascular endothelial cells, astrocytes, pericytes, and the basement membrane. The gap for the transport of substances across the BBB is 150–240 nm in rodents and 370–420 nm in humans [8], which is a limiting factor for diffusion and transport of therapeutic agents. It has been shown that almost 98% of drugs cannot cross the BBB to achieve effective therapeutic concentrations [9]. Consequently, for the treatment of stroke with classical drugs, it becomes necessary to use high doses that have a negative effect on healthy organs and tissues. At the same time, nanoparticles are able to penetrate into the brain not only by diffusion but also by active transport. To enhance the active transport of nanoparticles into the brain, their modification with receptor ligands is used: transferrin receptor, insulin receptor, low-density lipoprotein receptor (LDLR), angiopep-2 receptor, and the receptor for advanced glycation end-products (RAGE) [10,11].

Nanotechnology takes advantage of materials at the atomic and molecular level and involves the design, synthesis, and development of applications of nanomaterials. Nanomaterials for biomedicine can be of different shapes (spheres, dots, tubes, dendrites, and rods) and have different charges (neutral, positively, or negatively charged) [12]. Depending on the material type, shape, and charge, nanomaterials can have different characteristics, among which the following are key for stroke therapy: the size that helps to overcome the blood–brain barrier, stability of nanoparticles, duration of stay in the blood flow, and the ability to enter into metabolism and have an antioxidant effect [13]. Among the many nanoparticles, only four have pronounced antioxidant properties: cerium, aurum, platinum, and selenium. In addition, only nanoselenium enters the metabolism, and its nanoparticles regulate numerous functions of brain cells through the activity of selenoproteins [14,15,16]. Nanomaterials are considered to be nanoparticles with a size of 1–500 nm since this is the size that allows cells to easily absorb nanoparticles. Moreover, the smallest nanoparticles have a larger surface area to volume ratio, which leads to an increased ability of the nanomaterial to interact with cells [17]. Doping nanoparticles with active compounds (drugs and antioxidants) or encapsulating them in nanoparticles protects drugs from degradation and leads to an increase in their half-life from serum [18,19].

Unfortunately, many neuroprotective drugs have demonstrated failed efficacy in the treatment of acute ischemic stroke. Therefore, the search for effective approaches and neuroprotective compounds is an urgent task in modern biomedicine. It is known that antioxidants, anti-inflammatory cytokines, and receptor activators [20,21] are able to suppress the global [Ca^2+^]_i_ increase in neurons and astrocytes. However, delivering these compounds in effective concentrations to brain cells across the BBB and avoiding the side effects of their high doses are significant limiting factors for widespread use in the clinic. Therefore, the analysis of the positive and negative properties of known nanoparticles is of great interest for nanomedical research in the field of neurobiology. This review focuses on the analysis of the available scientific results of nanoparticles aimed at protecting brain cells from ischemic damage. Particular attention in the review is paid to one of the most promising types of nanoparticles, nanoselenium, a source of selenium for the brain. These nanoparticles have not received adequate attention for quite a long time despite the fact that selenium is a vital microelement with a pleiotropic effect.

## 2. Damaging Factors of Ischemia

**Figure 1:**
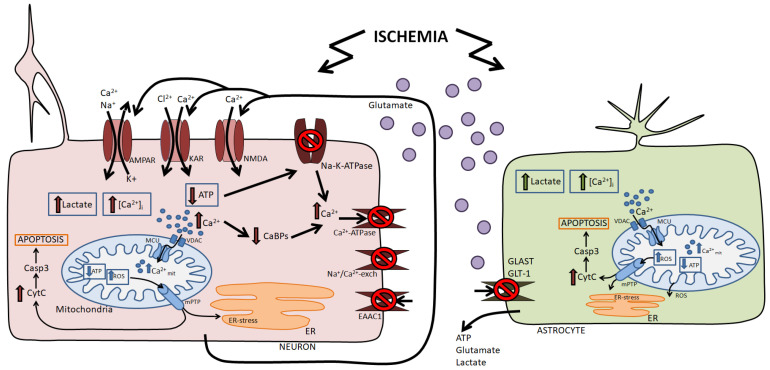
Damaging factors of ischemia on neurons and astrocytes. Briefly, a decrease in oxygen partial pressure and glucose content leads to a decrease in mitochondrial ATP synthesis, neuronal energy deficiency, disruption of ion homeostasis due to inhibition of cytoplasmic ATPases, and the release of large amounts of glutamate. Glutamate activates excitatory ionotropic glutamate receptors, resulting in glutamate excitotoxicity. As a result, brain cells experience a global irreversible increase in [Ca^2+^]_i_, Ca^2+^ overload of mitochondria, increased ROS production, ER stress, and activation of intracellular cell death pathways. Abbreviations: AMPAR—α-amino-3-hydroxy-5-methyl-4-isoxazolepropionic acid receptor, NMDAR—N-methyl-D-aspartate receptor, KAR—kainic acid receptor, [Ca^2+^]_i_—concentration of intracellular calcium ions, Na^+^/Ca^2+^-exch—sodium-calcium exchanger, EAAC1—excitatory (glutamate) amino acid transporter, ER—endoplasmic reticulum, mPTP—mitochondrial permeability transition pore, Ca^2+^_mit_—concentration of Ca^2+^ ions in mitochondria, ROS—reactive oxygen species, MCU—mitochondrial calcium uniporter, VDAC—voltage-dependent anion channels, ATP—adenosine triphosphate, CytC—cytochrome c, Casp3—caspase-3, GLAST—glutamate aspartate transporter 1, GLT-1—glutamate transporter 1. The figure was created using Microsoft PowerPoint (Microsoft Office 2016, Redmond, Washington, DC, USA).

Brain neurons are extremely sensitive to a lack of oxygen and glucose because they are an extremely active type of cell that consumes huge amounts of energy. In cells, the most energy-consuming processes are the functioning of ion pumps and the maintenance of ion homeostasis. In neurons, approximately 80% of ATP is spent on pumping ions and creating an ion gradient [22]. Therefore, hypoxia and ischemia, reducing the production of ATP by mitochondria, cause disturbances in the activity of ion exchangers and ion transporters. It has been shown that when ATP levels decrease by 50%, neurons depolarize, and the ability to maintain membrane potential is impaired due to the suppression of Na^+^/K^+^-ATPase activity [23]. This primary disruption of the key neuronal ATPase leads to the inhibition of Ca^2+^ ATPase and the Na^+^/Ca^2+^ exchanger and the influx of Ca^2+^ ions into the cytosol. As a result, Ca^2+^ homeostasis is disrupted (Figure 1).

In addition to the external Ca^2+^ ions influx, a pathological increase in [Ca^2+^]_i_ may occur due to impaired transport of cytosolic Ca^2+^ into intracellular pools [24] due to depletion of the capacity of intracellular calcium-binding proteins [25]. In brain cells, Ca^2+^ ions play an important role in the regulation of vital functions, e.g., the secretion of transmitters, excitability, synaptic plasticity, and gene transcription. An increase in [Ca^2+^]_i_ and loss of ionic homeostasis cause the release of large concentrations of glutamate, which leads to the phenomenon of glutamate excitotoxicity. One of the key mechanisms of glutamate release by neurons is the Ca^2+^-dependent activation of the calcium-binding protein synaptotagmin, which is located on the membranes of vesicles in the axon terminal and leads to the fusion of the vesicles with the membrane and the release of glutamate into the synaptic cleft [26]. Glutamate, in turn, activates excitatory glutamate receptors, NMDA receptors, AMPA receptors, and kainate receptors, which further enhances the increase in [Ca^2+^]_i_ (Figure 1). This cascade of events leads to postsynaptic death of brain cells. Moreover, the constant presence of glutamate leads to constant depolarization and, consequently, to a continuous influx of Ca^2+^ ions, creating a “vicious circle” and the release of a new portion of glutamate [27,28]. The accumulation of intracellular Ca^2+^ stimulates a number of catabolic processes due to the activation of Ca^2+^-dependent proteases, phospholipases, protein kinases, plasmogens, guanylate cyclase, NO synthase, and endonucleases [29,30].

To a certain extent, neurons are able to resist Ca^2+^ influx into the cytosol due to its storage in mitochondria and the endoplasmic reticulum (ER), but an increasing lack of ATP also leads to mitochondria Ca^2+^ overload and dysfunction. Mitochondria, under normal conditions, are ROS generators, but the antioxidant systems of neurons are able to resist this phenomenon. However, mitochondrial dysfunction leads to increased ROS production and rapid depletion of neuronal antioxidant systems, leading to oxidative stress. Additional sources of free radicals in brain cells during ischemia are Ca^2+^-dependent activation of NO synthetase, cyclooxygenase, and lipoxygenase [31]. Disruption of ion homeostasis, excessive increase in [Ca^2+^]_i_, and mitochondrial dysfunction lead to the activation of apoptosis (Figure 1). When apoptosis is activated by intracellular factors (intrinsic or mitochondrial apoptosis), the outer mitochondrial membrane is permeabilized, and signaling cascades are launched, leading to the activation of caspase-3 and -9 (Casp-9, Casp-3). Activation of the internal mechanism of apoptosis occurs due to a shift in the balance of pro- and anti-apoptotic intracellular factors towards apoptosis. Pro-apoptotic proteins of the Bax, Bak, Bad, Bid, etc., families interact with mitochondria and cause the release (through the mitochondrial pore, mPTP) of pro-apoptotic factors, cytochrome C, AIF, Smac/DIABLO, EndoG, and HtrA2/Omi, into the cytosol. Cytochrome C (cytosolic), procaspase-9, APAF-1, and dATP form the apoptosome, which is required for caspase-9 activation. Activated caspase-9 further activates effector caspases 3 and 7. Their action is balanced by the anti-apoptotic proteins Bcl-2 and Bcl-xL. In this case, external apoptosis (receptor-activated) occurs as a result of TNFα superfamily activation and is associated with the activity of caspase-9 and -3 [32,33,34]. Thus, the mechanisms of internal and external apoptosis activation are linked to caspases, which in turn are divided into initiator caspases (caspases-8 and 9) and effector caspases (caspase-3 and 7). Initiator caspases activate effector caspases, which are responsible for the final stage of apoptosis, DNA fragmentation, and phagocytosis of apoptotic bodies. The mutual enhancement of these two mechanisms has also been shown, and activated caspase-8 forms the tBid (truncated Bid) form from the pro-apoptotic protein Bid, which can be integrated into the outer membrane of mitochondria and promote the release of cytochrome C and other cytosolic factors, i.e., an internal mechanism for triggering apoptosis [35] (Figure 1).

Under ischemic conditions, in addition to programmed cell death (apoptosis), the necrotic process is activated (Figure 1). Cell death by this type of necrosis is associated with a violation of the plasma membrane integrity, degradation of organelles, swelling and vacuolization of the cell, condensation, and nonspecific degradation of DNA. In addition to the factors listed above, the induction of necrosis involves the hyperactivation of poly-(ADP-ribose) polymerase (PARP), an enzyme that completes ADP-ribose. Activation of PARP can be caused by cellular damage and results in rapid consumption of its substrate β-NAD^+^. During the resynthesis of β-NAD^+^, intensive consumption of ATP occurs, which can lead to necrosis due to lack of energy. Necrosis is regulated by kinases RIPK1 and RIPK3, as well as Omi proteases and some caspases. The signaling cascades responsible for the activation of apoptosis and necrosis are interconnected since kinases RIPK1 and RIPK3 are involved in the activation of caspases [36].

Less attention has been paid to the role of endoplasmic reticulum stress on brain cell damage during ischemia. ER stress, a molecular pathophysiological process, is accompanied by the accumulation of misfolded proteins in the ER, which initiates the unfolded protein response (UPR) aimed at restoring ER homeostasis. Activation of the UPR mitigates protein misfolding as it attenuates protein synthesis, enhances protein degradation, and activates target genes involved in restoring proteostasis [37]. Under ER stress, both adaptive and pro-apoptotic signaling pathways can be activated. The UPR signaling pathway consists of three key proteins, PERK, IRE1 and ATF6, the regulatory domains of which are normally associated with the BiP protein (BiP/GRP78) and are located in the ER lumen. However, under stress conditions, BiP dissociates, and the UPR protein triad is activated [37]. Interestingly, during the initial stages of ischemia, rapid activation of the PERK/p-eIF2α/ATF4 pathway occurs, leading to the inhibition of protein synthesis and activation of genes involved in antioxidant defense through the selective translation of activating transcription factor 4 (ATF4) [38]. However, prolongation of ER stress and long-term inhibition of protein synthesis activates the transcription factor CHOP, which is a trigger for increased expression of pro-apoptotic genes [39]. Unlike episodes of hypoxia, ischemic effects are more severe and prolonged, and ER stress leads to apoptosis rather than adaptive responses of brain cells.

Thus, during a stroke, branched pathological cascades are activated, triggered by a decrease in the partial pressure of oxygen and glucose levels. At each stage of ischemic signaling, there is an increase and intensification of cytotoxic effects and activation of vicious circles of reactions leading, at best, to programmed cell death, but in most cases to necrotic processes. The search for effective molecules capable of penetrating the blood–brain barrier and inhibiting cell death signaling pathways is an extremely urgent task of modern biomedicine.

## 3. Nanoparticles for Brain Protection

### 3.1. Nanoparticles as Regulators of Cellular Redox Status

The range of nanoparticles produced and studied in the context of the problem of strokes is quite wide. Nanoparticles made of metals and metal oxides, which primarily include nanoparticles of cerium oxide (CeNPs), aurum (AuNPs), and platinum (PtNPs), are known. Both of these types of nanoparticles are ROS-scavenging and have catalytic activity, imitating the properties of superoxide dismutase (SOD) and catalase (CAT) and converting •OH into O_2_ [14,15,40]. It has been shown that AuNPs and CeNPs can act as both antioxidants and pro-oxidants. Additionally, the toxic effect of AuNPs is determined by their diameter, when 20 nm AuNPs can reduce cerebral infarction in rats, while 5 nm AuNPs lead to enlarged infarction [41]. CeNPs have a neuroprotective effect and suppress OGD or H_2_O_2_-induced ROS production in a narrow concentration range of approximately 10 μg/mL, while higher concentrations of nanoceria induce ROS production by astrocytes [42].

Carbon-based nanoparticles, which are highly stable and are most often presented in the form of fullerenes and carbon nanotubes, have been used. Fullerenes (C60 nanoparticles) are spherical in shape, have abundant conjugated double bonds, and have the ability to absorb electrons. Therefore, they can perform the same function as SOD and scavenging free radicals [43]. The electrospun nanofiber scaffold, modified with 10 nm AuNPs, promoted immature neurons to grow axons more than branched trees [44]. Carbon-based nanoparticles are also easy to modify and easily adsorb active compounds on the surface. Carbon-based nanoparticles also exert a neuroprotective effect against oxidative stress and reduce the volume of cerebral infarction by 50% [45]. Fullerene nanoparticles activate the c-Jun NH_2_ terminal protein kinase (JNK) in the brain microvascular endothelial cells and inhibit the cleavage of polyADP-ribose polymerase (PARP) to inhibit cell apoptosis [46]. At the same time, carbon nanotubes are characterized by a significant limitation: they are not biodegradable in the body and easily form large aggregates.

Liposome nanoparticles are composed of amphiphilic molecules similar to biological membranes, which have also been used. Due to their properties, this type of nanoparticle is characterized by good biocompatibility and biodegradability, which allows them to be used as a shuttle for transporting active substances to the brain [47]. Polymeric nanoparticles are the most commonly used nanomaterials in drug delivery and are praised for their excellent biocompatibility and biodegradability. Polymeric nanoparticles are made of natural polymers (e.g., chitosan) or synthetic polymers (e.g., poly(lactic-co-glycolic acid) (PLGA), polylactide (PLA), poly(amidoa-mine) (PAMAM), or poly(methyl methacrylate) (PMMA)), and these materials have great surface modulation potential and good pharmacokinetic characteristics [48]. However, liposome nanoparticles and polymeric nanoparticles have limitations: an expensive production protocol, a relatively short “lifetime”, and the complexity of the process of their stabilization. It has been shown that polystyrene nanomaterials are changed from a sphere to a disk, with lower cell uptake and little impact on cell functions, such as cellular ROS generation [49].

Of particular interest are nanoparticles obtained from selenium (Se), which belongs to a class of lanthanides and is a non-metal. Most Se compounds, organic and inorganic, are easily absorbed from food and transported to the liver, the main organ of Se metabolism. A large number of methods can be used to synthesize selenium nanostructures, such as sonochemical synthesis, hydrothermal method, electrodeposition, physical adsorption via gas phase diffusion, laser ablation of a massive target, etc. [16,50,51]. Nanostructures of various shapes: trigonal, nanorods, nanoribbons, hexagonal prism, nanoplates, nanotubes, and spheres are obtained from selenium [52]. SeNPs, like nanoparticles of other origins, can enhance the effectiveness of ionized drug materials, improve the transport of water-soluble drugs, peptides, and many proteins, siRNAs, miRNAs, DNAs, i.e., used as nanotransporters of drugs to the brain. For selenium nanoparticles, it was shown that their modification with monoclonal antibodies (OX26) led to the activation of antioxidant systems of brain cells during ischemia, suppression of inflammation, and apoptosis [53]. At the same time, there are studies that demonstrate that SeNPs, without modification, are capable of activating protective signaling pathways, so the need for such active particles with additional molecules remains questionable. It was found that selenium activates transcriptional factors TFAP2C and SP1 to enhance GPx4 expression. In the bleeding brain stroke model, a single dose of selenium enters the brain, and it can promote the expression of the antioxidant GPx4 protein and protect the neurons [54].

### 3.2. The Effect of the Shape and Diameter of Nanoparticles on Their Cytoprotective Properties

The most important characteristics of nanoparticles that determine their effectiveness are their diameter and size. It is known that the use of nanoparticles for emergent large vessel occlusion has significant limitations. It has been shown that very small-sized nanoparticles (<10 nm) can rapidly clear through glomerular filtration and will be excreted, whereas too large-sized nanoparticles could impede their transport into the clot, carrying thrombolytic agents, or their transport of neuroprotective agents to the penumbra [55,56]. There are also results obtained from studies of the cytotoxic effects of nanoparticles, which demonstrate that small nanomaterials have greater activity but act within a few hours. For example, aurum nanoparticles are non-toxic with a diameter of 15 nm and are used as an effective nanotransporter for active compounds, but with a diameter of less than 5 nm (0.8–1.8 nm), they have an extremely cytotoxic effect [57]. The effects of nanoparticle shape and size have been explored to some extent in cancer cells. Thus, on cell lines A549, HepG2, MCF-7, and CGC-7901, it was shown that 5 nm-sized AgNPs are more toxic compared to 20 nm and 50 nm, causing a significantly more pronounced release of lactate dehydrogenase [58,59]. At the same time, there are practically no studies on “healthy” cells, including brain cells. It was found that silver nanoparticles (AgNPs) with a size of less than 50 nm showed a decrease in the percentage of living human mesenchymal stem cells after incubation for 1 h with a concentration of 10 μg/mL, while the use of 100 μg/mL nanoparticles with sizes of 10 and 20 nm does not affect the survival of progenitor human adipose-derived stem cells, which normally differentiate even after 24 h incubation [60,61]. There is convincing evidence that selenium nanoparticles with a diameter of a micrometer or more are biologically inert [62], while subnanomolar nanoparticles, on the contrary, are extremely toxic to cancer cells [63]. Indeed, SeNPs with a diameter of 36 nm are more bioavailable to eukaryotic cells than selenite or selenomethionine. When exposed to SeNPs of this diameter, the activity of glutathione peroxidases and thioredoxin reductases increases, providing an antioxidant effect [63]. In this regard, we conducted a comprehensive study, which showed that the use of SeNPs 50 nm-sized and 400 nm-sized was accompanied by an increase in the expression of necrosis and apoptosis genes, e.g., TRAIL, Cas-1, Bax, Bcl-xL, Nf-kb, and Tnfa. Therefore, SeNPs of this diameter are less effective as protectors against oxygen-glucose deprivation and reoxygenation. At the same time, 100 nm-sized SeNPs, causing Ca^2+^ oscillations without increasing the basal level of [Ca^2+^]_i_, increased the expression of protective genes while suppressing pro-apoptotic and pro-necrotic genes [64].

As for the shape of nanoparticles, there is even less research in this area compared to the mechanisms of the influence of the diameter of nanoparticles on the functions of nerve cells. Research in this area is focused on determining the circulation time of large filament-shaped nanoparticles in the bloodstream. It is precisely this physical feature that suggests the prospects of their use in therapy. It has been established that the residence time of long rod nanoparticles and short rod silica nanoparticles in the gastrointestinal system is significantly higher compared to spherical nanoparticles [65,66]. It is believed that the nanofilament may promote neuronal attachment and enhance the rate of neurite outgrowth [67,68]. It has been established that carbon nanotubes are structurally very similar to some elements of the neural network, and in the future, they can be used to modulate neuronal activity [69]. It has been found that carbon nanotubes can activate the electrical activity of neurons [70,71], suppress reactive astrogliosis [72], and modulate ion channel activity [73]. Selenium nanorods have, according to some parameters, more pronounced cytoprotective characteristics than spherical selenium nanoparticles. We found that selenium nanorods inhibit pro-inflammatory and pro-apoptotic signaling pathways during ischemia/reoxygenation, acting through the regulation of astrocyte calcium signaling. Selenium nanorods induce basal reactivity of A2-type astrocytes and enhance astrogliosis after ischemia [74], whereas spherical SeNPs do not induce reactive astrogliosis without ischemia [75].

Thus, despite a sufficient number of existing nanomaterials that can be used to prevent or treat strokes, there remain major problems in understanding the mechanisms of their action due to insufficient information on the dependence of the cytoprotective effectiveness of nanoparticles on their shape and size, and there are also limitations for a number of nanoparticles used in the form of their toxic effect on healthy organs and tissues. In this vein, nanoselenium has an undeniable priority, which is obtained from a vital microelement, selenium, which enters into the metabolism and acts through a separate class of proteins, selenoproteins and selenium-containing proteins, which will be discussed further.

## 4. Role of Selenium and Selenoproteins on Neurodegeneration in the Brain

Being an important trace element in animals and humans, Se plays a key role in maintaining normal physiological functions of the brain and has a neuroprotective effect. A feature of the brain that distinguishes it from other tissues is its ability to maintain selenium metabolism even with its deficiency [76,77]. Of particular interest are the epigenetic effects of dietary selenium sources. There remain large gaps in this area of research that require comprehensive and careful investigation. In this vein, Se is known to alter DNA methylation globally and at specific gene regions or loci through histone modification. Se-dependent signaling pathways have also been shown to influence nuclear proteins associated with epigenetic mechanisms through nucleosome remodeling, transcription, or DNA repair. Epigenetic effects of selenium sources in protecting brain cells from stroke may involve histone deacetylase 9 (HDAC9), which leads to an increase in HIF-1 and a reduction in Sp1 protein levels by deacetylation and deubiquitination. This effect of HDAC9 results in the downregulation of the anti-ferroptotic GPX4 gene, encoding the important selenium-containing protein glutathione peroxidase 4 [78,79]. Inhibition of HDAC9, including through selenium sources, may serve as one of the strategies for protecting brain cells. The neuroprotective effect of selenium is determined by its physicochemical properties, which allow it to enter into the metabolism and suppress oxidative stress, regulate the activity of ion channels, and inhibit apoptosis [80]. A source of selenium in the form of sodium selenite in the brain can activate a signaling cascade aimed at mitochondrial biogenesis, suppress ROS production under the influence of glutamate or hypoxia, and support mitochondrial functions and ATP synthesis during ischemia by inhibiting monoamine oxidase [80,81]. At the same time, organic and inorganic sources of selenium poorly penetrate the blood–brain barrier and are significantly cytotoxic [82,83]. Meanwhile, selenium nanoparticles as a source of selenium are free from these limitations [62].

Selenium realizes its functions through selenoproteins and selenium-containing proteins, the disruption of whose expression leads to neurodegenerative diseases. Selenoproteins are unique mammalian proteins because they contain residues of the selenium-containing amino acid selenocysteine and have a whole range of diverse functions, from antioxidant and immunomodulatory to the regulation of the processes of death and survival of body cells [49,84,85]. In humans, 25 selenoproteins have been identified, most of which are oxidoreductases involved in maintaining optimal cellular antioxidant status. Selenoproteins are found in the cytosol, mitochondria, and nucleus; a separate large group is expressed in the endoplasmic reticulum, and these selenoproteins are called ER-resident selenoproteins.

There are two types of glutathione peroxidases expressed in the brain: GPX1 (in neurons and astrocytes) and GPX4 (in neurons) (Table 1). GPX4 is localized both in the cytoplasm of neurons and in the nucleus and mitochondria. The key role of these selenium-containing proteins is to protect brain cells from oxidative stress [86]. The second type of selenium-containing proteins are thioredoxin reductases (TNXRs), which are involved in the formation of reduced disulfide bonds in cells and play an important role in maintaining redox balance through the utilization of hydroperoxide. Brain tissue expresses TXNRD1 (cytosol) and TXNRD2 (mitochondria) [87]. When TXNRD1 or TXNRD2 is knocked out, early mouse embryonic death occurs [84]. A homozygous mutation in human TXNRD2 has been shown to cause familial glucocorticoid deficiency without a cardiac phenotype. Interestingly, TXNRD mutations in mice exhibit more severe phenotypes than the corresponding human phenotypes. This raises the question of whether some TXNRD variants can compensate for the loss of other TXNRDs in humans but not in mice. Thus, TXNRDs, as representatives of antioxidant enzymes, contribute not only to the antioxidant system but also to cell proliferation and apoptosis [49,88].

Methionine sulfoxide reductase (MSR, SELENOR) is an antioxidant enzyme with reductase activity responsible for the repair of methionine-oxidized proteins and restoring their functions. In brain cells, it is expressed in the cytoplasm. It can reduce the content of methionine sulfoxide generated by methionine residue oxidized by ROS [89]. The functions of this protein are poorly studied, and it does not directly cause neurodegenerative processes [84,90]. However, by analogy with methionine sulfoxide reductase A, it is still assumed that selenium deficiency may reduce the activity of SELENOR and increase the content of methionine sulfoxide, promoting the formation of Aβ small fiber oligomer to accelerate the development of cognitive impairment [91] (Table 1).

SELENOW is expressed in the cytoplasm of brain cells and is a protein responsible for redox reactions. SELENOW is most highly expressed in the cerebral cortex, fascia dentata, and hippocampus [49,92].

SELENOP is a key selenium transporter in the brain, and its expression is predominantly detected in the cytosol of neurons. However, at the same time, it was revealed that SELENOP is formed and secreted by cultured astrocytes in response to neuronal activation and, accordingly, astrocytes can serve as an intracerebral source of selenium for neurons, controlling its availability [49]. In addition to the transport function, SELENOP has GPx-like enzymatic activity due to the presence of the redox motif in its composition. SELENOP, through its redox motif, is involved in the reduction in phospholipid hydroperoxides in the presence of glutathione, playing an important role along with GPX and TXNRD in antioxidant defense and being a redox regulator in vivo [93] (Table 1).

**Table 1:** Non-ER-resident selenoproteins and brain damage.

	Physiological Action	Effects of Protein Disruption	Ref.
GPX1	Antioxidant action. Intracellular hydrogen peroxide utilization. Overexpression of GPX1 improves the differentiation of mouse embryonic stem cells into neural stem cells and dopaminergic neurons.	GPX1 gene knockout does not affect the normal development of mice. Knockout may exacerbate tissue damage if mice are subjected to brain damage using toxins or limiting cerebral blood flow.	[94,95,96]
GPX4	Antioxidant action. GPx4 is the only enzyme that utilizes glutathione. GPx4 is the only GPX that can utilize membrane phospholipid hydroperoxides as its substrate, reducing phospholipids and cholesterol hydroperoxides. Protects neurons from death during ferroptosis through inhibition of lipid peroxidation.	The knockout causes embryonic lethality, and conditional GPX4 knockout mice exhibit cognitive disruption and hippocampal neurodegeneration. Mutations in the GPX4 gene cause spondylometaphyseal dysplasia sedagati type in children. Neuron-specific knockout causes astrocyte hyperproliferation and neuroinflammation.	[97,98,99,100,101,102]
TXNRD1	Antioxidant action. Catalyzes electron flux from NADPH through TrxR to Trx, which then keeps cellular biomolecules (proteins, lipids, and DNA) in the reduced form.	Nervous system-specific inactivation leads to ataxia and tremors that are associated with cerebellar hypoplasia. Neuron-specific gene deletion leads to age-related neurodegeneration and impaired neuronal development. Conditional ablation of TXNRD1 in neuronal progenitors reveals only a mild cerebellar defect.	[49,87,103]
TXNRD2	Antioxidant action. Participation in the regulation of proliferation. Inhibition of apoptosis.	A homozygous mutation in human TXNRD2 results in glucocorticoid deficiency without a cardiac phenotype. Nervous system-specific Txnrd2 knockout mice do not show any neurological abnormalities. Constitutive gene inactivation is embryonic-lethal.	[87,104,105]
Methionine sulfoxide reductase (MSRB1, SELENOR)	Responsible for the reduction in methionine sulfoxide. Involved in the regulation of synaptic plasticity by reducing oxidized CaMKIIα and CaMKIIβ in mice.	Does not cause neurodegeneration. Spatial memory and learning deficit, along with an upregulation of GFAP in MSRB1 deletion. Not directly shown; however, knockout of the methionine sulfoxide reductase A gene leads to neurodegenerative diseases, increased phosphorylation of the TAU protein (microtubule-associated protein), and loss of integrity of astrocytes and increased Aβ precipitation. It is likely that disruption of SEKENOR expression may lead to a similar effect. Spatial memory and learning deficit, along with an upregulation of GFAP in MSRB1 deletion. Not directly shown; however, knockout of the methionine sulfoxide reductase A gene leads to neurodegenerative diseases, increased phosphorylation of the TAU protein (microtubule-associated protein), loss of integrity of astrocytes, and increased Aβ precipitation. It is likely that disruption of SELENOR expression may lead to a similar effect.	[84,90,97,106]
SELENOW	Antioxidant action.	Knockout leads to increased H_2_O_2_-induced apoptosis of cortical neurons.	[49]
SELENOP	Transport of selenium into the brain. Antioxidant action. Modulatory effects in mesolimbic dopaminergic signaling. Exogenous SELENOP prevents the release of dopamine vesicles. Detoxification functions through the binding and inactivation of heavy (copper and cadmium) and transition metals (mercury and iron).	Ataxia. Epilepsy. Disruption of long-term potentiation. Loss of Parvalbumin interneurons. Reactive astrogliosis. Hippocampal neurogenesis is reduced. Depletion of SELENOP and its receptor ApoER2 results in spatial memory impairment in mice as well as defects in synaptic transmission. SELENOP-deficient mice exhibit selenium deficiency in the brain and myelin sheath abnormalities in the brainstem. Genetic deletion of SELENOP results in increased release of dopamine vesicles in response to methamphetamine.	[84,85,86,87,88,107,108,109,110,111,112]
SELENOI	Participation in myelin biosynthesis. Maintaining phospholipid homeostasis.	Inactivation of the gene in mice is embryonic-lethal. The SELENOI mutation causes atrophy of the cerebellum and brainstem, which can cause sensorineural deafness, blindness, and seizures. Homozygous missense mutations in SELENOI correlate with seizure activity in some individuals of a pedigree with hereditary spastic paraplegia.	[49,113,114]

Selenoprotein SELENOI–ethanolamine phosphotransferase catalyzes one phospholipid biosynthesis stage and makes a significant contribution to the neuronal network’s formation and the axon’s functioning [115].

Among the 25 selenoproteins, 7 proteins are ER-resident proteins: selenoprotein M (SELENOM), selenoprotein F (SELENOF), selenoprotein T (SELENOT), selenoprotein K (SELENOK), selenoprotein S (SELENOS), iodothyronine deiodinase 2 (DIO2), selenoprotein H (SELENOH), and selenoprotein N (SELENON) [116]. It has long been believed that the expression of selenoproteins in the brain is responsible for antioxidant defense. However, in recent years, it has become clear that the role of selenoproteins does not end with the antioxidant effect. ER-resident selenoproteins are involved in maintaining Ca^2+^ homeostasis of nerve cells, receptor-mediated neurotransmission, development of the inhibitory GABAergic system of the brain, protecting neurons from hyperexcitation and glutamate excitotoxicity, and inhibition of ferroptosis [117,118,119]. Among the ER-resident selenoproteins, the most expressed in the brain are SELENOM, SELENOF (Sep15), SELENOT, SELENOK, SELENOS, and SELENON, whereas DIO2 is expressed to the least extent and selectively in specialized brain regions (Table 2). The key functions of these selenoproteins are the regulation of Ca^2+^ homeostasis of the ER, participation in protection against oxidative stress, and control of protein folding, and many of their functions remain unknown. Impaired expression of ER-resident selenoproteins is associated with the induction of ER stress, inflammation, and apoptosis [120]. Of all the types of deiodinases, only DIO2 is expressed in brain cells. The function of DIO2 is to convert T4 to active T3. DIO2 has been shown to be mainly found in glial cells and deiodinates T4 and T3. The unambiguous function of DIO2 in the brain has not yet been determined; most likely, DIO2 stabilizes the homeostasis of thyroid hormones in the brain. Most ER-resident selenoproteins are predominantly expressed in neurons, but their expression has also been shown in astrocytes. Moreover, under pathological conditions, the level of expression of a number of selenoproteins increases in astrocytes [119]. In the brain, disruption of the functions and expression of ER-resident selenoproteins causes a number of neurodegenerative processes, which are summarized in Table 2.

**Table 2:** ER-resident selenoproteins and brain damage.

	Physiological Action	Effects of Protein Disruption	Ref.
SELENOM	Participation in maintaining ER and cytosolic Ca^2+^ homeostasis. Overexpression of SELENOM in neurons reduces H_2_O_2_-induced [Ca^2+^]_i_ increase.	Knockout leads to an increase in [Ca^2+^]_i_, probably due to its leakage from the ER, activation of oxidative stress, and apoptosis. In neurons overexpressing presenilin 2 (PS2), Ca^2+^ efflux from the ER was correlated with decreased SELENOM expression.	[49,121,122,123,124,125,126]
SELENOF	Control of N-glycosylated proteins folding through its interaction with UDP-glucose-glycoprotein glucosyltransferase. Participates in the secretion of some glycoproteins. Involved in adaptive ER stress. In response to moderate ER stress under the action of tunicamycin, SELENOF expression increases, and brain cells adapt.	A powerful stressor effect on the ER using DTT leads to a decrease in SELENOF expression and induction of apoptosis. Mice with SELENOF knockout were viable and fertile, with normal brain morphology and no activation of endoplasmic reticulum (ER) stress. Oxidative stress was elevated in the livers, and prominent nuclear cataracts developed at an early age. The expression of SELENOF mRNA was downregulated in the hippocampus and substantia nigra of a Parkinson’s mouse model.	[127,128,129,130]
SELENOT	Control of protein processing in the ER. Possessing oxidoreductase activity, it participates in the antioxidant protection of cells. Catalyzes redox reactions with thiol groups of thiol-disulfide oxidoreductases (ERp57 and protein disulfide isomerase) and various chaperones (BiP, calnexin, calreticulin, and glucose-regulated protein GRP94). Regulation of the protein N-glycosylation. Regulation of the Ca^2+^ ions pool in the ER. Regulation of dopaminergic neurotransmission (increased dopamine levels) through increased tyrosine hydrolase activity.	Mice with a neuron-specific knockout of SELENOT exhibit decreased volumes of the hippocampus, cerebral cortex, and cerebellum. Perturbation of SELENOT expression induces apoptosis in neurons during the postnatal period. Suppression of expression leads to increased ROS production and nitric oxide, depletion of the ER Ca^2+^ pool, disruption of hormone secretion, and activation of UPR signaling.	[49,88,120,131,132,133]
SELENOS	Anti-apoptotic effects. Participates in the folding and degradation of misfolded proteins associated with the ER (ERAD process). Overexpression increases the resistance of astrocytes to ER stress and inflammatory stimuli.	Suppression of expression correlates with astrocyte death. Gene knockout results in brain cell apoptosis mediated by ER stress.	[120,133,134]
SELENOK	Participation in the ERAD pathway of protein degradation in the ER. Participation in the restoration of the cell membrane bilipid layer. Regulation of brain cells Ca^2+^ homeostasis. Involvement in synaptic neurotransmission through functional interaction between SELENOK and NMDAR. Activation of IP3R and [Ca^2+^]_i_ increase in microglia through the interaction of SELENOK with palmitoyltransferase (DHHC6). Activating microglial migration and phagocytosis to suppress brain neuroinflammation.	The knockout leads to the disruption of intracellular Ca^2+^ homeostasis and the functioning of synaptic glutamate receptors. Knockout results in an imbalance in the expression of NMDAR subunits in neurons and neurodegeneration.	[49,116,135]
SELENON	Protecting cells from oxidative stress. Regulation of Ca^2+^ homeostasis through interaction with the ryanodine receptor RYR1. Neutralizes the inhibitory effect of hydroperoxide on SERCA2b.	Not found.	[136,137,138]
DIO2	Stabilizes brain thyroid hormones homeostasis. It is expressed predominantly in astrocytes, but through neuroglial interactions, it can regulate the neuronal network activity.	Impaired motor control. Leads to anxiety.	[139,140,141,142]

Despite the relevance and high significance of research into the mechanisms of brain cell protection from ischemia, there are only a few articles that show a direct connection between the effect of ischemia and selenoprotein activity. For ER-resident selenoproteins, it has been shown that brain cells respond to transient focal ischemia by increasing the expression of SELENOS. This increase in SELENOS expression promotes brain cell survival by suppressing inflammation. The mechanism of this SELENOS action involves the activation of astrocytes [143]. As for other selenoproteins, only SELENOP [49], whose functions in the brain are the most studied, are available in the brain to protect from ischemia. Thus, it is obvious that selenoproteins are involved in neurodegeneration and most likely play a purely cytoprotective role. However, the mechanisms that involve selenoproteins in protecting brain cells from ischemia have been virtually unexplored. These gaps in understanding hinder the active use of selenium sources for stroke prevention.

## 5. Selenium Nanoparticles in Protecting Cells from Ischemic Factors

### 5.1. Possible Signaling Pathways for Cytoprotective Action of Selenium Nanoparticles

As shown above, in recent years, increasing attention has been paid to studying the possibility of using nanoparticles in neurological diseases. Oxidative stress is a key factor in the pathogenesis of neurological diseases, especially cerebral ischemia, so the use of nanoparticles with antioxidant effects is a promising strategy for neuroprotection. Of particular interest in this area are selenium nanoparticles (SeNPs), which are low-disperse bioactive compounds with powerful antioxidant abilities, high bioavailability, and reduced toxicity compared to other Se-containing compounds. SeNPs are able to cross the blood–brain barrier, accumulate in the brain, and prevent apoptosis [144,145].

**Figure 2:**
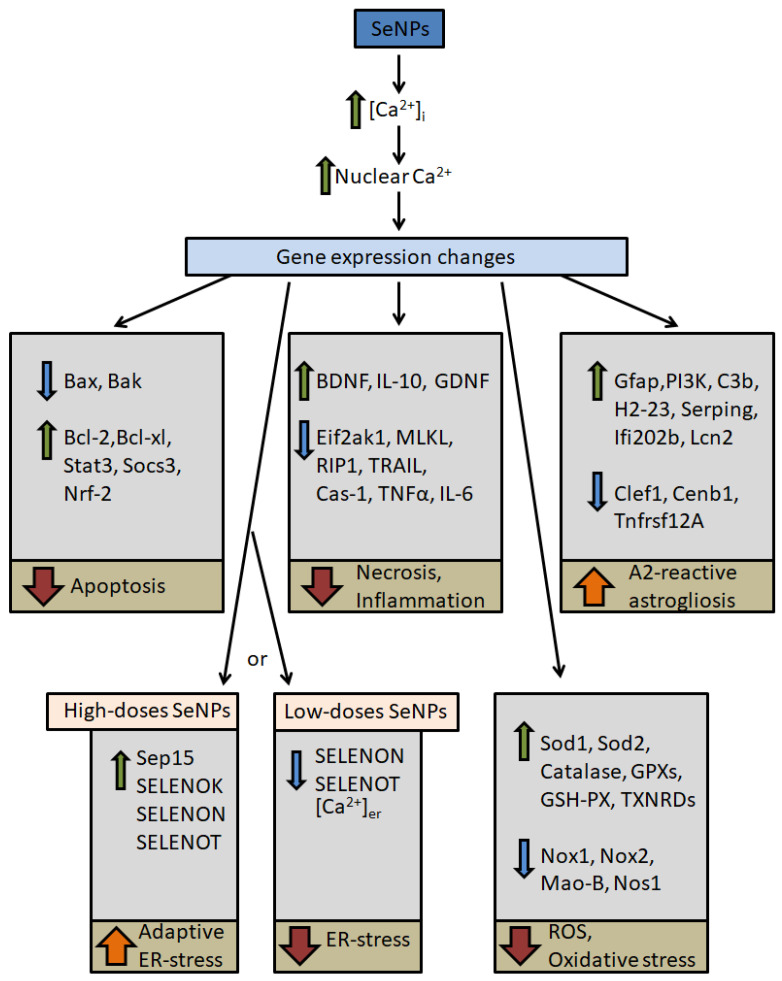
Chronic cytoprotective effects of selenium nanoparticles in the brain under ischemia/reoxygenation. The figure was created using Microsoft PowerPoint (Microsoft Office 2016, Redmond, Washington, DC, USA).

The protective effects of SeNPs are known in neurodegenerative diseases: Alzheimer’s disease, Parkinson’s disease, Huntington’s disease, and amyotrophic lateral sclerosis. In Alzheimer’s disease, SeNPs inhibit Aβ aggregation, combat oxidative stress, and lessen tau protein hyperphosphorylation, promoting neuronal survival [146,147,148]. In Parkinson’s disease, nanoselenium improves behavior abnormalities and prevents the loss of dopaminergic neurons [149]. Similarly, in Huntington’s disease, SeNPs act by attenuating oxidative stress and inhibiting the aggregation of Huntington proteins [150]. A common effect of SeNPs in these neurodegenerative diseases is the suppression of oxidative stress. In cerebral ischemia, it was found that the use of SeNPs led to increased expression of antioxidant enzymes (Sod1, Sod2, Catalase, GPXs, GSH-PX, and TXNRDs) and suppression of the expression of pro-oxidant proteins (Nox1, Nox2, Mao-B, and Nos) (Figure 2). This effect of SeNPs enhanced the antioxidant status of brain cells and protected them from ROS production and oxidative stress during ischemia/reoxygenation [151,152,153]. The antioxidant effect of SeNPs can also be exerted through an increase in the expression of brain-derived neurotrophic factors BDNF and GDNF (Figure 2) [154,155,156]. Enhancing BDNF expression in neurons through the inhibition of oxidative stress protects the most ischemia-sensitive populations of GABAergic neurons, contributing to the preservation of the functional state of neuronal networks [157,158]. Oxidative stress with depleted antioxidant systems of cells leads to the activation of cell death pathways, involving a branched signaling cascade. Selenium-containing agents have been shown to inhibit apoptosis through increased BCL-2 expression and decreased Bax levels [159,160,161]. The anti-apoptotic effect of using nanosized selenium particles was also aimed at suppressing the expression of caspase-3 through IκB-α degradation and NF-κB nuclear translocation [162,163]. In the cortical cells, selenium nanoparticles [74,151,156] led to the suppression of cell death from damaging factors of ischemia and reoxygenation through the activation of key protective protein (Bcl-2, Bcl-xL, Socs-3, IL-10) expression and suppression of proteins responsible for the activation of apoptosis and necrosis pathways (Caspase-1, Rip1, TRAIL, Tnfα, etc.) (Figure 2). In addition, many selenoproteins and selenium-containing proteins are known as antioxidant and antiapoptotic proteins in brain cells. SeNPs have been found to lead to increased selenoprotein expression [164,165]. We established the dose-dependent effect of selenium nanoparticles in cortical cells when high concentrations of SeNPs led to the adaptive ER stress activation, and low SeNPs concentrations inhibited ER stress; in both cases, cells were protected from death during ischemia (Figure 2). High doses of SeNPs (2.5–10 µg/mL) led to apoptosis, suppressing necrosis, which was accompanied by an increase in caspase-3 activity, but at the same time, there was an increase in the expression of the anti-apoptotic genes Bcl-2, Bcl-xL, redox homeostasis genes Nrf2 and Socs3, and suppression of the proapoptotic marker Bax expression. This shift from necrotic to programmed apoptotic cell death during ischemia/reoxygenation was due to increased expression of Sep15 (SELENOF), SELENOK, SELENON, and SELENOT, causing adaptive ER stress. A low dose (0.5 µg/mL) of SeNPs suppressed ischemia-induced necrosis and apoptosis. This was accompanied by a decrease in proapoptotic protein expression, a decrease in caspase-3 activity, and a decrease in the level of ER selenoproteins, but at the same time, an increase in the anti-apoptotic protein’s expression was observed. An increase in cell survival may indicate that low SeNP doses under normal physiological conditions improve the condition of brain cells, and under toxic conditions, they can protect cells, i.e., this dose can be prophylactic or, moreover, provide preconditioning of brain cells (Figure 2). This protective effect of SeNPs is associated with a decrease in selenoprotein SELENOK, SELENON, SELENOT, and SELENOP expression, reducing ER stress. Suppression of ER stress was accompanied by a decrease in the level of Ca^2+^ ions in the ER and redistribution of Ca^2+^ in cells, which is also closely related to the preconditioning of nerve cells [166,167]. In addition, it has been shown that changes in gene expression are also regulated by intracellular Ca^2+^ dynamics and increases in Ca^2+^ ions in the nucleus regulate the expression process [25,168]. In addition, the global increase in Ca^2+^ ions in the cytosol of neurons and astrocytes during ischemia is one of the key causes of cell death (Figure 1). Disturbances in Ca^2+^ homeostasis activate either programmed cell death (apoptosis) or collapse due to loss of control over cell death mechanisms (necrosis) [28]. There are suggestions that SeNPs may help maintain Ca^2+^ homeostasis in neurons by enhancing the expression of parvalbumin [169,170]. Since parvalbumin is not only a marker of GABAergic neurons but also a key protein responsible for their survival under hypoxia/ischemia [171], nanoselenium likely contributes to the preservation of neuronal network inhibition during ischemia. On the other hand, as indicated above, nanoselenium affects the expression of ER-resident selenoproteins involved in the regulation of intracellular Ca^2+^ ion concentration. It was found that the incubation of cortical astrocytes with SeNPs for 24 h leads to the depletion of the Ca^2+^ pool of the ER, and this depletion of the Ca^2+^ pool is correlated with the activation of proteins responsible for the activation of adaptive ER stress [172]. However, these mechanisms require detailed study.

A particular effect of SeNPs is the activation of reactive astrogliosis. Reactive astrogliosis is a process of complex molecular, cellular, and functional changes in astrocytes in response to brain damage. It is customary to subdivide astrogliosis into A1 and A2 types. Changes in the protein’s expression and, accordingly, the functions of astrocytes towards the A1-phenotype of astrogliosis lead to the loss of astrocyte functions and activation of cell death pathways, while reactive A2-type astrogliosis activates the protective functions of astrocytes [173,174]. Various nanomaterials are known to be capable of activating reactive astrogliosis [175,176,177]. For SeNPs, it was found that the A2 type of reactive astrogliosis is activated predominantly, which is characterized by increased expression of a number of proteins (Figure 2) that perform cytoprotective functions during ischemia/reoxygenation [149,178,179].

Thus, selenium nanoparticles activate the Ca^2+^ signaling system in brain cells, increasing the concentration of cytosolic calcium and, as a result, an increase in Ca^2+^ ions in the nucleus induces changes in gene expression (Figure 2). As a result, anti-apoptotic and anti-inflammatory signaling pathways are activated, the antioxidant status of brain cells is enhanced, A2-reactive astrogliosis is activated in astrocytes, and, depending on the concentration, ER stress is suppressed or adaptive ER stress is triggered. As a result, brain cells become “prepared” for the damaging factors of ischemia/reoxygenation and are able to survive under these conditions. Studies show that cytoprotective changes induced by SeNPs persist after ischemia. At the same time, there are no studies that studied the effects of SeNPs on the restoration of brain tissue after a stroke, including the formation of new neuronal connections, the migration of progenitor cells, etc.

### 5.2. Acute Effects of Selenium Nanoparticles

**Figure 3:**
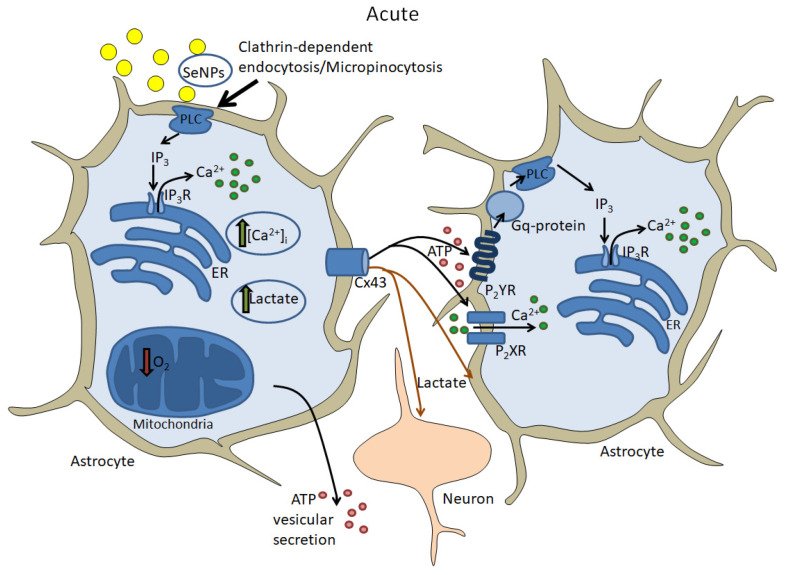
Acute effects of selenium nanoparticles on neurons and astrocytes. The figure was created using Microsoft PowerPoint (Microsoft Office 2016, Redmond, Washington, DC, USA).

The effects of selenium nanoparticles aimed at changing gene expression and regulating (inhibiting) cell death in the brain have been described, effector proteins have been identified, and some signaling cascades of their cytoprotective action have been identified. Changes in genome expression take time, and many data indicate that this change is related to the activation of the cellular Ca^2+^ signaling system. However, the rapid effects of SeNPs on brain cells have been virtually unexplored. We found that the application of SeNPs in an acute experiment causes a dose-dependent increase in [Ca^2+^]_i_ [74] in astrocytes obtained from various regions of the brain. At the same time, an increase in [Ca^2+^]_i_ is not recorded in neurons, but a clear neuroprotective effect of SeNPs, which is associated with neuroglial interactions, is revealed. Activation of the Ca^2+^ signaling system has also been shown for various cancer cell lines [180] and immune cells [181]. There is compelling evidence that SeNPs enter cells through endocytotic mechanisms. SeNPs have been shown to enter mouse brain cells in an in vivo model via transferrin receptor-mediated endocytosis, inhibit the inflammatory response, and increase the survival of hippocampal neurons [182]. We found that SeNPs, activating Ca^2+^ signals in astrocytes, penetrate into cells simultaneously through two endocytosis pathways: clathrin-dependent endocytosis and micropinocytosis (Figure 3). Along with active endocytosis, the action of nanoparticles also involves passive transport into cells, e.g., through passive penetration [183,184].

Endocytosis of SeNPs into the astrocyte activates phospholipase C (PLC) and triggers the phosphoinositide signaling cascade (Figure 3). Currently, there is information that under the lead oxide nanoparticles, the gene and protein expression of phospholipase PLCβ1 significantly increases in macrophages and liver cells [185]. However, such a change cannot be called a quick activation of the PLC. It can be assumed that nanoparticles, including nanoselenium, have extremely high reactivity [186] and can directly activate PLC. However, this hypothesis requires serious research. Activation of PLC leads to IP_3_-dependent mobilization of Ca^2+^ ions from the ER and an increase [Ca^2+^]_i_ in astrocytes. As a result, Ca^2+^-dependent release of ATP and lactate occurs through Cx43 connexin chemical channels into neighboring astrocytes and the extracellular environment [74]. Other authors have also shown the effects of selenium and nanoselenium on the expression and phosphorylation of Cx43 [187,188]. The result is paracrine activation of the entire astrocyte network and suppression of ischemia-induced neuronal hyperexcitation [74], likely due to lactate uptake by neurons. Since the protective effect of lactate released by astrocytes during ischemia/reoxygenation is well known [189,190]. A significant contribution to the protection of neurons and neighboring astrocytes can be made by the release of ATP, which, acting through the activation of P2Y and P2X purinergic receptors, can suppress ischemic neuronal hyperexcitation [191]. One of the putative mechanisms for the suppression of neuronal activity by ATP secreted by astrocytes is the activation of adenosine receptors by adenosine formed as a result of extracellular degradation of ATP [192]. In addition, the propagation of the Ca^2+^ wave in the astrocytic network under the action of SeNPs may contribute to a bigger release of gliotransmitters that suppress ischemia-induced neuronal hyperexcitation.

In addition to the fact that SeNPs contribute to a change in the expression of redox proteins towards antioxidant protection of brain cells from ischemia, nanoselenium alone or in combination with antioxidants can play the role of a ROS scavenger [193,194]. Applying SeNPs to cortical neuroglial cultures did not increase ROS production but led to the suppression of H_2_O_2_- or ischemia-induced ROS overproduction [151].

Thus, the rapid effects of nanoselenium involve neuroglial interactions, the trigger of which is the activation of the phosphoinositide signaling system of astrocytes and the mobilization of Ca^2+^ ions from the ER. An increase in [Ca^2+^]_i_ in astrocytes triggers paracrine activation of the entire astrocyte network and a Ca^2+^ wave, which induces the release of ATP, lactate, and probably other gliotransmitters, which leads to the inhibition of ischemic neuronal hyperexcitation.

## 6. Conclusions

The results of this review show the complexity of the damaging effects of ischemia on brain cells and the potential of nanotechnology for stroke prevention. It turned out that of the nanomaterial’s variety, only a small number of nanoparticles can be used to protect brain cells from ischemia since many materials are highly toxic in the form of nanoparticles. It was also discovered that nanoselenium is practically devoid of the disadvantages of other nanoparticles, and, most importantly, nanoselenium is a highly active and easily accessible source of selenium, a vital microelement. In the form of nanoparticles, selenium is able to overcome the blood–brain barrier and enter cellular metabolism as a regulator of the expression of not only selenoproteins but also a number of other protective proteins. This review shows all the known chronic (hours to days) and acute (minutes to tens of minutes) effects of selenium nanoparticles on brain cells and the probable mechanisms of action of these nanoparticles.

## Data Availability

The data presented in this study are available on request from the corresponding authors.
